# Interactive Effects of Firebreak Construction and Elevation on Species Diversity in Subtropical Montane Shrubby Grasslands

**DOI:** 10.3390/plants14223456

**Published:** 2025-11-12

**Authors:** Chengyang Hui, Yougui Wu, Qishi Liu, Zhangli Shui, Huihui Wu, Qian Cai, Weilong Zhou, Wenjuan Han, Mingjian Yu, Jinliang Liu

**Affiliations:** 1 College of Life and Environmental Science, Wenzhou University, Wenzhou 325035, China; 2 Qingyuan Conservation Center, Qianjiangyuan-Baishanzu National Park, Qingyuan 323800, China; 3 Ecological Forestry Development Center, Lishui Economic and Technological Development Zone, Lishui 323000, China; 4 Baishanzu Management Bureau, Qianjiangyuan-Baishanzu National Park, Lishui 323000, China; 5 College of Life Sciences, Zhejiang University, Hangzhou 310058, China

**Keywords:** firebreak, diversity, species composition, elevation effect, edge effect, shrubby grassland ecosystem, herb and shrub

## Abstract

Montane shrubby grasslands, as one of the world’s important ecosystems, are highly sensitive to climate change and human activities, especially in the subtropical regions experiencing rapid economic development. However, little is known about how anthropogenic activities, such as firebreak construction, interact with elevation to influence plant diversity in these ecosystems. Shrub and herbaceous communities were surveyed in subtropical montane shrubby grassland within Baishanzu National Park, eastern China. Nine transects were established along firebreaks, each with two edge plots near firebreak and two interior plots away firebreak, and twelve additional control plots in adjacent undisturbed areas. Species diversity was assessed using the Hill index. Our results revealed distinct responses of shrubs and herbs to firebreak disturbance and elevation. Firebreaks reduced shrub diversity but enhanced herb diversity, and both groups exhibited contrasting elevational patterns. In control areas, shrub diversity decreased while herb diversity increased with elevation, whereas in firebreak zones, these relationships were altered, with edge plots showing a hump-shaped diversity pattern. Differences in shrub diversity but not herbs between interior and edge plots decreased with elevation. Species composition also differed significantly between firebreak and control areas, driven mainly by elevation in control areas and by soil properties near firebreaks. These findings demonstrate that firebreak construction reshapes the elevation–diversity relationships of both herbs and shrubs, highlighting the sensitivity of high-elevation montane shrubby grasslands to small-scale disturbances. Effective firebreak management should therefore account for both elevational context and disturbance intensity to maintain ecosystem biodiversity and stability.

## 1. Introduction

Montane shrubby grassland ecosystems are widely distributed across major mountain ranges worldwide, including the Himalayas, Andes, and Alps, and represent one of the critical vegetation types in alpine zones [1,2,3]. These ecosystems primarily occur on steep ridges and slopes between the treeline and snowline, dominated by shrubs and herbaceous plants adapted to low temperatures, strong winds, and nutrient-poor soils [4], including species from the genera *Rhododendron*, *Salix* and *Carex*. Globally, montane shrubby grasslands provide critical ecological services, including regulating regional hydrological cycles, enhancing carbon sequestration capacity, and sustaining rich endemic biodiversity [5]. However, these ecosystems are highly sensitive to environmental changes. Even small elevational differences may substantially shape species diversity patterns [6,7,8]. Climate warming is driving their upward range shifts to higher elevations [9], while human activities (e.g., road construction, firebreak establishment, grazing and tourism) reduce native species richness and alter species composition through direct vegetation destruction and the introduction of alien species [10]. For example, on the Tibetan plateau, overgrazing has led to declines in native vegetation cover and exacerbated soil erosion [11], whereas in the Alps, soil disturbance caused by ski run construction have severely impaired the recovery of dwarf shrubs, particularly at high elevations [12]. Such disturbances can trigger cascading ecological effects, ultimately threatening the stability of montane shrubby grassland ecosystems [13]. This is especially true in subtropical regions, where these ecosystems are subject to more frequent human disturbances. Yet, research on montane shrubby grasslands in subtropical high-elevation areas remains scarce.

Firebreaks are typically strips or patches of land where flammable fuels have been removed to prevent the occurrence and spread of wildfires [14,15,16]. As key infrastructure for wildfire management, firebreaks can significantly alter surrounding plant communities [17,18]. Previous studies have shown that firebreak construction and associated activities can affect plant species richness and composition along firebreak edges [19]. For instance, Seipel et al. [20] demonstrated that soil disturbance from firebreak construction in grassland ecosystems significantly changed plant community composition, leading to declines in native herb cover and facilitating the spread of invasive species. The removal of trees during firebreak construction, coupled with increased human activities (e.g., tourist trampling and physical disturbance), also has substantial impacts on surrounding woody vegetation [21,22]. These disturbances not only directly reduce the woody plant survival but may also further shift plant species composition and diversity in the firebreak edge zones. However, most existing studies have concentrated on temperate grasslands or lowland forests, while the ecological impacts of firebreaks in subtropical montane shrubby grassland ecosystems remain largely understudied.

In addition to anthropogenic disturbances, elevation is a major factor shaping the diversity and community composition of montane plants by regulating climatic conditions, soil properties, and biological interactions [23]. While plant species diversity generally declines with elevation, it often peaks at mid-elevations [24]. This pattern is influenced by a combination of factors, including temperature, precipitation, UV radiation, and soil nutrient availability [25,26,27]. For example, low temperatures and short growing seasons at high elevations limit the growth of most plants [28], whereas middle elevations may provide more favorable thermal and hydrological conditions that support higher species diversity [5]. As elevation increases, harsher conditions such as low temperatures, strong winds, and nutrient-poor soils select for specialized species adapted to extreme environments, including dwarf shrubs and alpine meadow plants [29]. These stressors promote the evolution of adaptive traits such as reduced height and thickened leaves, leading to distinct vertical zonation in species composition along elevational gradients [30].

Although a hump-shaped pattern of species diversity with elevation is frequently reported, some studies have documented a monotonically decreasing diversity with increasing elevation [31,32,33,34], whereas others have reported higher diversity at greater elevations in certain regions [35,36]. Notably, these patterns are typically derived from areas with broad elevational ranges and are often associated with transitions between distinct ecological zones [37]. In high-elevation subregions, particularly at ecotones between shrublands and grasslands, ecosystems are subject to intensified environmental filtering. However, systematic investigations into how elevation shapes species diversity in these specific zones remain scarce.

In regions with high human activity, interactions between elevation and anthropogenic disturbances further influence plant diversity [38]. For example, firebreak construction has been shown to alter species diversity, with elevation gradients modulating the magnitude of these changes [39]. In lower elevation areas of high mountains, firebreak construction and maintenance have only limited effects on plant diversity at firebreak edges [40]. At higher elevations, harsher environmental conditions exacerbate the impacts of firebreak disturbances, further impeding community recovery and leading to reduced species diversity and compositional homogenization [41]. Moreover, microclimatic factors (e.g., wind speed and humidity) and nitrogen as well as carbon storage gradients across firebreak edges also shift with elevation, thereby influencing the strength and direction of edge effects [42,43]. Therefore, quantifying the interaction between elevation and anthropogenic disturbances is critical for predicting future dynamics of subtropical montane shrubby grassland ecosystems.

This study focuses on the shrubby grassland ecosystem at approximately 1800 m elevation within Baishanzu National Park (BNP), located in the subtropical region of eastern China. The park reaches a maximum elevation of 1929 m and encompasses a complete vertical zonation. At approximately 1800 m, firebreaks constructed along ridgelines form a continuous system locally known as the Qianba Line (denoting firebreaks situated around 1800 m). This zone is dominated by shrublands and shrubby grasslands characterized by typical subtropical montane species, including *Pinus hwangshanensis*, *Stranvaesia davidiana* var. *undulata*, *Rhododendron simsii*, *Molinia japonica* and *Miscanthus sinensis*, representing a typical subtropical montane shrubby grassland ecosystem. Areas below this zone are dominated by subtropical montane forests. In recent years, with the growing prominence of BNP, tourism activities utilizing the Qianba Line firebreaks as hiking routes have surged, accompanied by steadily increasing visitor numbers. Such human activities could lead to a series of ecological issues, including soil erosion, vegetation degradation, alteration of the diversity and composition of plant species, as well as the invasion of alien species and the spread of disturbance-tolerant native species [44,45,46]. We established nine transects along firebreaks, each with two edge plots and two interior plots, and twelve additional control plots in undisturbed areas. Species diversity was assessed using the Hill index (*q* = 0, 1, and 2), which give greater weight to common species as the parameter *q* increase. This study aims to investigate how elevation and firebreak influence plant species diversity and composition in this ecosystem, and whether soil physicochemical properties mediate these effects.

## 2. Results

### 2.1. The Difference in Species Diversity Among Plots

**Figure 1:**
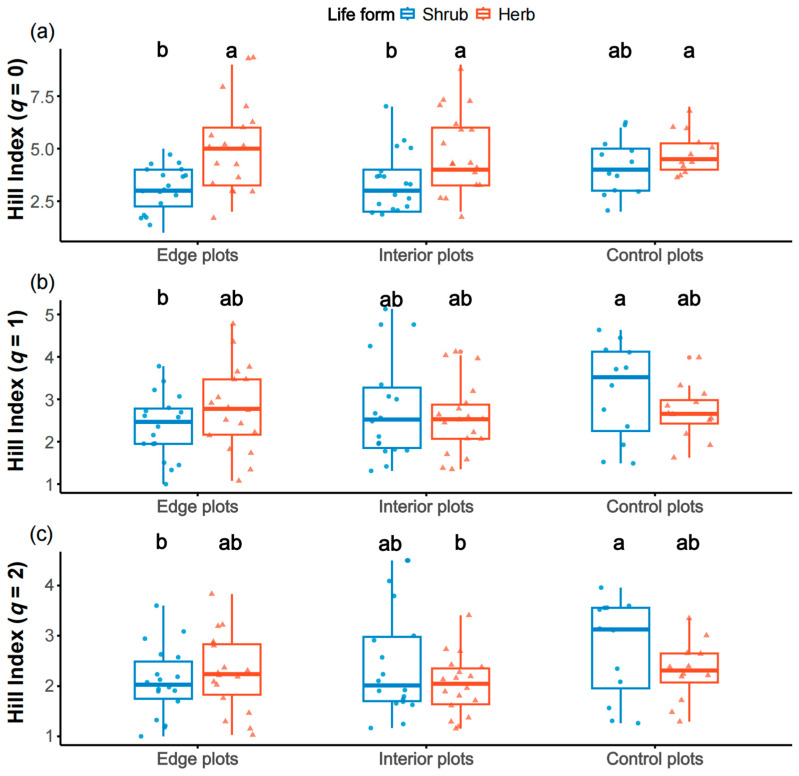
The significance difference in Hill indices with (**a**) *q* = 0, (**b**) 1 and (**c**) 2 for herb and shrub species between edge, interior, and control plots. Treatments with significant differences (*p* < 0.05) are labeled with different letters.

This survey recorded 49 vascular plant species, belonging to 28 families. The shrubs were dominated by *Rhododendron simsii* and *Stranvaesia davidiana* var. *undulata*, while the herbs were dominated by *Miscanthus sinensis* and *Molinia japonica.* When *q* = 0, the Hill index of herbs in edge, interior, and control plots was significantly higher than shrubs in both edge and interior plots (*p* < 0.05). When *q* = 1, the Hill index of shrubs in the control plots was significantly higher than that in the edge plots (*p* < 0.05). When *q* = 2, the Hill index of shrubs in the control plots was significantly higher than shrubs in the edge plots and herbs in the interior plots (*p* < 0.05) (Figure 1). The Hill indices (*q* = 0, 1, and 2) of both shrubs and herbs were not significantly associated with distance from the firebreak (see Supplementary Figure S1).

### 2.2. Species Diversity Responses to Elevation Across Firebreak and Control Areas

**Figure 2:**
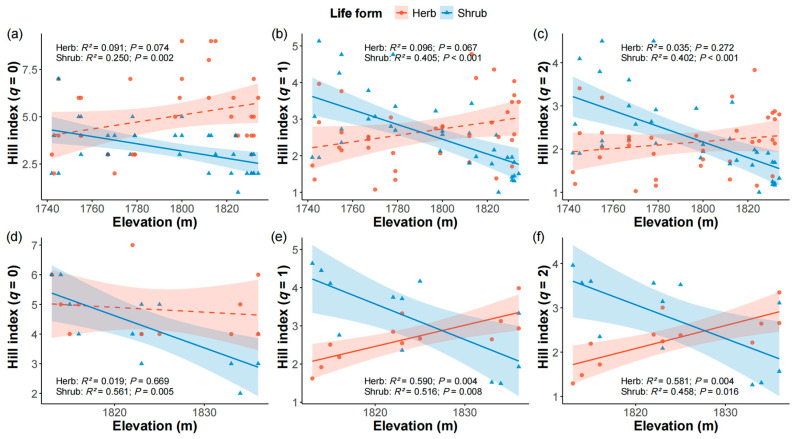
Relationships between Hill indices with *q* = 0 (**a**,**d**), 1 (**b**,**e**) and 2 (**c**,**f**) of herb (red) and shrub (blue) species and elevation in the firebreak areas (**a**–**c**) and control areas (**d**–**f**). The shaded areas represent 95% confidence interval of the linear regression models. Solid lines denote statistically significant relationships (*p* ≤ 0.05), whereas dashed lines indicate non-significant relationships.

The Hill indices (*q* = 0, 1, and 2) of shrubs significantly decreased with increasing elevation in both the firebreak and control areas (Figure 2). When *q* = 0, there was no significant relationship between elevation and Hill index of herbaceous species in firebreak and control areas (Figure 2a,d). The Hill indices of herbaceous species in the control plots increased significantly with elevation when *q* = 1 (Figure 2e, *R*^2^ = 0.590, *p* = 0.004) and *q* = 2 (Figure 2f, *R*^2^ = 0.581, *p* = 0.004), whereas no significant relationship was found between elevation and the Hill indices (*q* = 1, and 2) of herbaceous species in firebreak areas (Figure 2b,c).

### 2.3. Species Diversity Responses to Elevation Across Edge and Interior Plots

**Figure 3:**
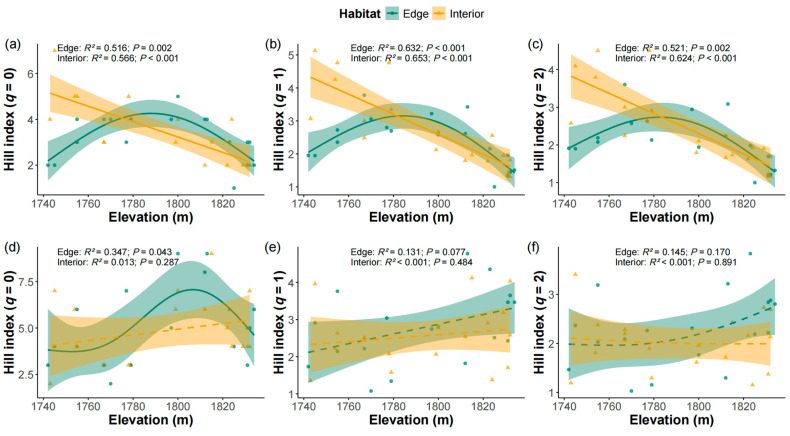
Relationships between Hill indices with *q* = 0 (**a**,**d**), 1 (**b**,**e**) and 2 (**c**,**f**) of shrub (**a**–**c**) and herb (**d**–**f**) species and elevation in the edge plots (green) and interior plots (yellow). The shaded areas represent 95% confidence interval of the generalized additive models. Solid lines denote statistically significant relationships (*p* ≤ 0.05), whereas dashed lines indicate non-significant relationships.

The Hill indices of shrubs in the interior plots decreased significantly with elevation when *q* = 0 (Figure 3a, *R*^2^ = 0.556, *p* < 0.001), *q* = 1 (Figure 3b, *R*^2^ = 0.653, *p* < 0.001), and *q* = 2 (Figure 3c, *R*^2^ = 0.624, *p* < 0.001). In contrast, The Hill indices of shrubs in the edge plots exhibited a hump-shaped pattern, increasing significantly with elevation up to 1790 m and then declining at higher elevations for *q* = 0 (Figure 3a, *R*^2^ = 0.516, *p* = 0.002), *q* = 1 (Figure 3b, *R*^2^ = 0.632, *p* < 0.001) and *q* = 2 (Figure 3c, *R*^2^ = 0.521, *p* = 0.002). For herbaceous species in the edge plots, the Hill index at *q* = 0 increased with elevation up to 1810 m, followed by a decline at higher elevations (Figure 3d, *R*^2^ = 0.347, *p* = 0.043). However, no significant relationships were found between elevation and the Hill indices at *q* = 1 and *q* = 2 (Figure 3e,f). In the interior plots, the Hill indices (*q* = 0, 1, and 2) of herbaceous species showed no significant relationship with elevation (Figure 3d–f).

### 2.4. Effects of Environmental Factors on Species Diversity

When evaluating the effects of elevation, topography, and soil physicochemical properties on species diversity, elevation showed a significant negative effect on the Hill indices (*q* = 0, 1, and 2) of shrub communities in edge, interior, and control plots, and was negatively associated with the Hill index (*q* = 0) of herbaceous communities in edge plots (Table 1). Soil pH exhibited highly significant positive effects on the Hill indices (*q* = 0, 1, and 2) of shrub communities in edge plots and significantly enhanced the Hill index (*q* = 0) of herbaceous communities in both edge and interior plots (Table 1). Total carbon (TC) was significantly positively correlated with the Hill index of herbaceous communities in edge plots (*q* = 0) and in control plots (*q* = 2). Total phosphorus (TP) was negatively correlated with the Hill index of herbaceous communities in edge plots (*q* = 0), but positively associated in control plots (*q* = 2); Available phosphorus (AP) showed significant positive correlations with the Hill indices of herbaceous communities in control plots (*q* = 1 and 2), whereas NH_4_^+^-N (ammonium nitrogen) exhibited significant negative effects on these indices (Table 1).

### 2.5. Diversity Differences of Edge and Interior Plots Along Elevation

**Figure 4:**
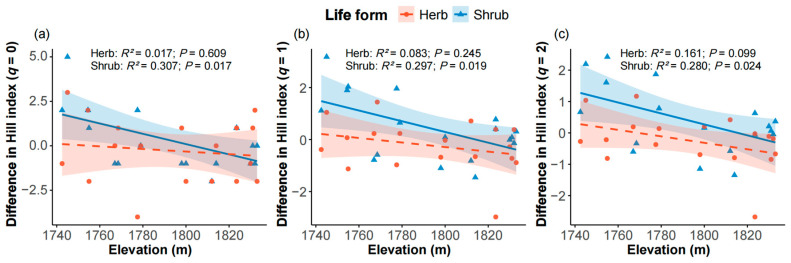
Differences in Hill indices with *q* = 0 (**a**), 1 (**b**) and 2 (**c**) between interior and edge plots for herb (red) and shrub (blue) species along elevation. The shaded areas represent 95% confidence interval of the linear regression models. Solid lines denote statistically significant relationships (*p* ≤ 0.05), whereas dashed lines indicate non-significant relationships.

The differences in Hill indices between interior and edge plots for shrubs decreased significantly with increasing elevation when *q* = 0 (Figure 4a, *R*^2^ = 0.307, *p* = 0.017), *q* = 1 (Figure 4b, *R*^2^ = 0.297, *p* = 0.019), and *q* = 2 (Figure 4c, *R*^2^ = 0.280, *p* = 0.024). However, the differences in Hill indices (*q* = 0, 1, and 2) for herbaceous species showed no significant relationship with elevation (Figure 4).

### 2.6. Species Composition and Environmental Drivers

**Figure 5:**
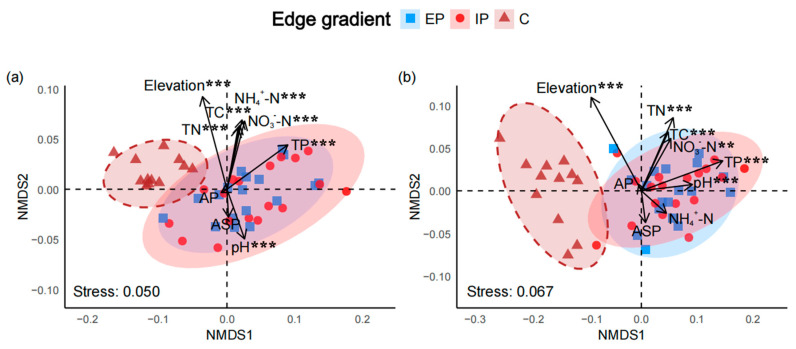
Non-metric multidimensional scaling (NMDS) analysis of shrub (**a**) and herb (**b**) species composition based on Bray–Curtis distances. Ellipses represent 85% confidence intervals for edge gradients in firebreak zones (EP: Edge plots; IP: Interior plots) and non-firebreak zones (C: Control plots). Asterisks indicate the significance predictor factors (** *p* ≤ 0.01; *** *p* ≤ 0.001).

The species composition of shrubs (Figure 5a, PERMANOVA test: EP vs. C, *R*^2^ = 0.550, *p* = 0.001; IP vs. C, *R*^2^ = 0.490, *p* = 0.001) and herbs (Figure 5b, PERMANOVA test: EP vs. C, *R*^2^ = 0.719, *p* = 0.001; IP vs. C, *R*^2^ = 0.657, *p* = 0.001) of edge plots and interior plots in the firebreak areas significantly differed with the control areas. However, neither shrubs nor herbs composition showed significant differences across edge gradients within firebreak areas (Figure 5; see Supplementary Table S1). Elevation, TN, TC, NO_3_^−^-N, NH_4_^+^-N, pH and TP were found to significantly affect the species composition. Specifically, elevation primarily influenced species composition in area without firebreaks, while the remaining topography, and soil physicochemical properties primarily influenced species composition in the firebreak areas (Figure 5; see Supplementary Table S2).

## 3. Discussion

Firebreak construction and management, as forms of intensive anthropogenic disturbance, significantly alter soil nutrient availability and physicochemical properties [19,47,48]. These disturbances along firebreak edges may function as environmental filters, favoring plant species that are tolerant of disturbance. Such changes influence species diversity and community composition by modifying resource availability and soil conditions [19,49]. In this study, a comparative analysis was conducted among plots located near firebreaks (edge plots), those located further away (interior plots), and control plots without firebreaks. We examined alpha diversity, the relationship between species diversity and elevation, and differences in species composition among these plots. Our findings reveal that the firebreak construction at high elevations exerts differential impacts on herbaceous and shrub diversity of species, particularly altering the pattern of the relationship between species diversity and elevation. We also found that soil physicochemical properties significantly influenced species diversity and composition, but microclimatic factors (e.g., soil temperature and moisture) that may affect species diversity were not considered, representing a limitation of this study, and future studies will integrate these factors.

Within the firebreak areas, although no significant differences were observed in the Hill indices of herbaceous and shrub communities between edge and interior plots, comparisons with the control plots revealed that, with increasing *q* values, the Hill indices of shrub communities in control plots were significantly higher than those in edge plots (Figure 1; see Supplementary Figure S1), consistent with the PERMANOVA results for shrubs (Figure 5a). This indicates a higher diversity of common shrub species in control plots, suggesting that firebreak construction disproportionately affects common shrub species. These results support the notion that periodic anthropogenic disturbances promote herbaceous dominance while disadvantaging slow-growing woody species [50].

Regarding the relationship between species diversity and elevation, we found that herbaceous Hill indices were not significantly correlated with elevation in the firebreak areas (Figure 2a–c), whereas, in control areas, herb diversity at higher *q* value increased significantly with elevation (Figure 2d–f). According to the interspecific competition hypothesis, environmental gradients can reduce dominance by competitive species, thereby facilitating species coexistence [51,52,53]. Therefore, the higher diversity of common species at higher elevations in control areas are likely due to reduced competitive dominance. In this study, dominant species such as *Miscanthus sinensis* and *Molinia japonica* declined in abundance with increasing elevation, likely weakening their suppressive effects and facilitating greater herb coexistence at higher elevations.

In contrast, the absence of significant correlations between herbaceous diversity and elevation in firebreak areas suggests that firebreaks may weaken the elevational response of herbaceous communities. This could be attributed to disturbances associated with firebreaks, such as surface exposure and increased susceptibility of soils to rainfall erosion, which in turn may disrupt the natural elevational gradient in soil nutrient distribution (TN: *R*^2^ = 0.411; TC: *R*^2^ = 0.274; TP: *R*^2^ = 0.877; AP: *R*^2^ = 0.003; NH_4_^+^-N: *R*^2^ = 0.106; NO_3_^−^-N: *R*^2^ = 0.254). These disturbances likely reduce the elevation-dependent positive effects of soil properties—particularly TC, TP, and AP—on common species (Table 1).

Unlike herbs, shrubs diversity declined significantly with elevation in both firebreak areas and control areas (Figure 2), indicating that shrub species are more sensitive to elevation. Additionally, shrubs are generally less efficient than herbs in acquiring and utilizing nutrients such as TC, TP, AP, and NH_4_^+^-N (Table 1), resulting in a competitive disadvantage at higher elevations [54]. These factors collectively contribute to the observed decline in shrub diversity with increasing elevation. Overall, our results suggest that while firebreak may weaken the elevational diversity patterns in herbaceous communities, shrub diversity remains strongly influenced by elevation in montane shrubby grassland ecosystems.

We further observed contrasting elevation-diversity patterns between edge and interior shrub plots in the firebreak area. Shrub diversity in edge plots followed a hump-shaped pattern with elevation, whereas diversity in interior plots declined linearly with elevation (Figure 3a–c). Both patterns have been reported in natural environments [55,56]. In edge plots, anthropogenic disturbances such as tourist trampling and physical disturbance were more pronounced, especially at lower elevations, leading to reduced shrub diversity along the firebreak. The greater shrubs diversity observed at mid-elevations may be attributed to moderate disturbance levels associated with firebreaks that promote species coexistence, supporting the intermediate disturbance hypothesis [57]. At higher elevations, increased environmental filtering likely contributed to the decline in shrub diversity. Conversely, in interior plots where anthropogenic disturbance was minimal, the lack of coexistence-promoting mechanisms may have resulted in a steady decline in diversity with elevation.

Interestingly, herbaceous diversity in edge plots, when considering rare species (*q* = 0), also exhibited a hump-shaped pattern with elevation, with the highest value of 9 observed at mid-elevation (Figure 3d), suggesting that moderate disturbance at mid-elevations may facilitate the occurrence of rare species. Rare species are often more sensitive to certain soil physicochemical properties [58,59], such as pH and TC, which had a positive effect on the coexistence of rare species in the edge plots (Table 1). In contrast, the Hill indices (*q* = 1, and 2) of herbs showed no significant elevation-related trends in either edge or interior plots (Figure 3e,f), indicating that common herbaceous species were less affected by firebreak-induced edge effects. These findings further highlight the differential sensitivity to of shrubs and herbs to environmental stress, with common herbaceous species displaying greater adaptability to changes in resource availability at higher elevations.

Moreover, the differences in shrub diversity (*q* = 0, 1, and 2) between interior and edge plots decreased significantly with increasing elevation, whereas the corresponding differences in herbaceous diversity were not significant (Figure 4). These results suggest that firebreaks have a more pronounced impact on shrub species diversity at lower elevations, while herbaceous species exhibit stronger adaptability and are less sensitive to interactions between firebreaks and elevation.

In terms of species composition, no significant differences were found between edge and interior plots for either herbs or shrubs (Figure 5; see Supplementary Table S1), consistent with the finding of Weeks et al. [60], suggesting that the firebreak-induced edge effects do not significantly alter community composition at the local scale. However, when comparing species composition in plots adjacent to firebreaks with that in control plots, significant differences were observed for both herbs and shrubs (Figure 5; see Supplementary Table S1). While such differences may be partly attributed to spatial isolation and variation in local species pools, species composition in firebreak plots was more strongly associated with soil variables—such as TN, TC, NO_3_^−^-N, NH_4_^+^-N, pH, and TP—compared to control plots, where species composition was more closely related to elevation (Figure 5; see Supplementary Table S2). These differences may be largely due to firebreak-induced alterations in soil physicochemical properties [61].

## 4. Materials and Methods

### 4.1. Study Site

The study was conducted above 1700 m elevation along the Qianba Line firebreak area within Baishanzu National Park (BNP) in eastern China (27°32′25″–27°58′28″ N, 118°57′49″–119°22′9″ E). The firebreak, initially a 1–2 m footpath, was expanded in 1987 to ~12 m width for fire prevention. Since then, it has been maintained annually through the manual removal of vegetation and flammable materials. Such management measures, which aim to prevent the spread of forest fires by building fireproof trails and removing plants from them, are currently the main measures adopted in subtropical regions. Over time, the firebreak has also become a hiking route, attracting a growing number of visitors due to its accessibility and panoramic mountain views. The Qianba Line hiking trail derives its name from crossing eleven peaks above 1800 m and is low largely co-located with firebreaks constructed along the ridge lines, serving as the main infrastructure for tourism in the region. The vegetation at the site mainly consists of species such as *Rhododendron simsii*, *Stranvaesia davidiana* var. *undulata*, and *Miscanthus sinensis*, forming a typical subtropical montane shrubby grassland. This typical subtropical montane shrubby grassland ecosystem is mainly distributed in areas above 1700 m in elevation in the park, generally distributed from 1700 m to 1900 m. In the area below 1700 m, subtropical forests are mainly distributed, including Evergreen Broadleaf Forest, Mixed Evergreen and Deciduous Broadleaf Forest, Deciduous Broadleaf Forest, Mixed Needleleaf and Broadleaf Forest, and Coniferous Forest.

The BNP is characterized by a subtropical maritime monsoon climate, receiving an average annual precipitation of 1667.8–1804.6 mm and 1578.9–1699.8 h of sunshine per year [62]. Despite its geographic location within the subtropical zone, the region’s high elevation results in a relatively cool mean annual temperature of 18.2–18.3 °C [63]. Soil types vary along the elevational gradient, with zonal red soils dominating lower altitudes and yellow soils prevailing at higher elevations [64].

### 4.2. Study Design and Plant Community Survey

**Figure 6:**
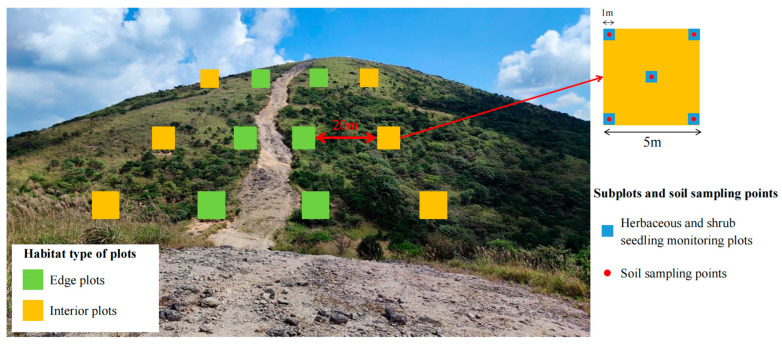
Example of paired sampling plots established on both sides of the firebreak along the “Qianba Line” in Baishanzu National Park.

To investigate the effects of firebreak construction and elevation on species composition and diversity in subtropical shrubby grassland ecosystems, we selected a representative section of the Qianba Line firebreak area and established nine transects oriented perpendicular to the firebreak along elevation, with an elevation range of 1700 m to 1850 m, where the vegetation at the mountain peak is highly sensitive to even small elevational changes. Along each transect four 5 m × 5 m plots were established. Two plots were established near the firebreak on opposite sides (<5 m from the firebreak), designated as the edge plots (EP). The other two plots were positioned farther away on opposite sides of the firebreak (>25 m from the firebreak), designated as the interior plots (IP) (Figure 6). A total of 36 plots were established across nine transects. In addition, to assess the impact of firebreak construction on plant diversity and composition, a comparative study was also conducted in areas without firebreak but at similar elevation within BNP, and the distance from the site in the firebreak area is approximately 6 km. Using the same sampling design, 12 additional plots were established as control plots (C). For the vegetation survey, the species names, life form, and heights of herbaceous and shrubs were recorded within each plot. Shrub individuals with a basal diameter (BA) greater than 1 cm or a height greater than 1.3 m were tagged and geo-referenced. The diameter at breast height (DBH) was measured for shrubs with a height greater than 1.3 m, whereas the BA was measured for shrubs with a height less than 1.3 m but with a BA greater than 1 cm. Within each 5 m × 5 m plot, five 1 m × 1 m subplots were set up at the four corners and center. In these subplots, herbaceous species and shrubs with a BA less than 1 cm were recorded for species names, life forms, abundance, and average height.

### 4.3. Environmental Factor Survey

To assess the influence of environmental factors on species diversity and composition across different life forms (herbs vs. shrubs) and treatment groups, we measured several topographic and soil physicochemical variables for each plot. This included elevation, distance from the firebreak, aspect (ASP), total phosphorus (TP, mg/kg), total nitrogen (TN, %), total carbon (TC, %), available phosphorus (AP, mg/kg), ammonium nitrogen (NH_4_^+^-N, mg/kg), nitrate nitrogen (NO_3_^−^-N, mg/kg), and soil pH. Elevation was recorded using a handheld GPS device: Garmin GPSMAP 64s (Garmin Ltd., Olathe, KS, USA). Aspect was measured with a compass: Brunton TruArc 20 (Brunton Inc., Riverton, WY, USA), then standardized using the formula of −cos((2πASP)/360) to make the maximum value at South and the minimum value at North. Soil samples were collected from the 5 m × 5 m plot using a five-point sampling method (four corners and center), with surface soil (0–10 cm) collected after removing litter, stones, roots, and debris. The homogenized soil was then passed through a 2 mm mesh and stored at −20 °C for subsequent analysis of soil physicochemical properties. Soil TN and TC contents were determined using an elemental analyzer: vario MACRO cube (Elementar Analysensysteme GmbH, Langenselbold, Germany). TP was extracted using H_2_SO_4_ and HClO_4_, and analyzed by inductively coupled plasma optical emission spectrometry: ICP-OES Optima 8300 (PerkinElmer Inc., Waltham, MA, USA). AP was extracted using a 0.03 mol/L NH_4_F-0.1 mol/L HCl solution, and also determined with ICP-OES. NH_4_^+^-N and NO_3_^−^-N were extracted with 2 mol/L KCl solutions and determined using a continuous flow analysis system: San++ (Skalar B.V., Breda, Holland). Soil pH was measured in a 1:2.5 soil-to-water suspension. Specifically, 10.00 g of air-dried soil was mixed with 25 mL of CO_2_-free deionized water, stirred intermittently for 30 min, and allowed to settle for another 30 min, and measured with Mettler-Toledo S20 pH meter (Mettler-Toledo, Greifensee, Switzerland) [65].

### 4.4. Statistical Analysis

We calculated the Hill indices using the following formula:

(1) Dq=∑i=1Spiq11−q

where *S* is the total number of species, *p_i_* is the relative abundance of the species *i* (*i* = 1, 2, …, *S*) [66]. The order of *q* determines the sensitivity of the index to species abundances, balancing emphasis between richness and evenness [67]. In which *q* = 0 represents species richness, assigning equal weight to all species and emphasizing rare species; *q* = 1 gives weights proportional to species abundance, emphasizing common species; and *q* = 2 gives more wight to dominant species, reducing the influence of rare species [68,69].

For the following analyses, all plant species were classified by life form into herbs and shrubs. Hill indices of herbs and shrubs in each plot were calculated using the *hillR* package (R v.4.4.1) [70]. To quantify differences in Hill indices of herbs and shrubs among treatment groups (Edge plots, Interior plots, and Control plots), we used one-way analysis of variance (ANOVA) followed by the least significant difference (LSD) post- hoc tests. Linear regression models were used to test the relationships between Hill indices with *q* = 0, 1, and 2 of herb and shrub communities and elevation in firebreak areas and control areas. We also analyzed the relationship between elevation and the differences in Hill indices between interior and adjacent edge plots (i.e., the Hill index of herbs or shrubs in the interior plot minus that in corresponding edge plots, calculated separately for the left and right sides of each transect), to assess how elevation influences disparities in species diversity between edge and interior habitats.

To analyze how firebreak edge and interior conditions influence the elevational patterns of species diversity in herbs and shrubs., we applied generalized additive models (GAMs) using the *mgcv* package (R v.4.4.1) by modeling relationships between Hill indices with *q* = 0, 1, and 2 of shrubs and herbs and elevation for edge plots and interior plots separately [71]. GAMs require selecting an appropriate distribution family and link function based on the response variable. Since Hill indices are continuous and generally approximately normally distributed, we applied a Gaussian distribution with an identity link function [72].

To identify key environmental drivers of species diversity, we performed a model selection using the second order Akaike Information Criterion which adjusts for small samples (AICc) [73,74]. We first constructed linear regression models (LMs) including candidate environmental predictors (Elevation, ASP, TP, TN, TC, AP, NH_4_^+^-N, NO_3_^−^-N and soil pH). An exhaustive model selection was then performed the “*dredge*()” and “*get.models*()” functions in the *MuMIN* package (R v.4.4.1). The model with the lowest AICc was selected as the best-fit model, and the slope estimates, significance levels (*p*-values), and coefficients of determination (*R*^2^) were extracted to evaluate the contribution of each predictor.

To test how firebreak influence shrub and herbaceous species composition, we conducted non-metric multidimensional scaling (NMDS) with Bray–Curtis dissimilarities with the “*metaMDS*()” function in the *vegan* package (R v.4.4.1) across edge plots and interior plots in firebreak areas and non-firebreak area. Permutational multivariate analysis of variance (PERMANOVA) was performed using the “*adonis2*()” function in *vegan* package (R v.4.4.1) to test for significant differences in species composition among treatment groups [75]. Environmental variables were fitted to the NMDS ordination using the “*envfit*()” function, and their associations with ordination axes were tested via 999 permutations.

All analyses were conducted using R v.4.4.1 (R Core Team, Vienna, Austria).

## 5. Conclusions

This study demonstrates that firebreak construction significantly influences plant diversity and community structure in montane shrubby grasslands, with differential effects on herbaceous and shrub species. Firebreak construction can significantly reduce the diversity of shrub species. While firebreaks weakened the elevation–diversity relationship for herbaceous communities, and shrub diversity remained strongly constrained by elevation. Additionally, distinct elevation-diversity patterns emerged between edge and interior plots, with moderate disturbance at mid-elevations promoting the coexistence of shrub and rare herbaceous species. Although firebreaks did not significantly alter species composition between edge and interior plots, they did induce community-level changes relative to control plots, largely mediated by shifts in soil nutrient conditions. These findings highlight the importance of considering both elevation and disturbance intensity in firebreak planning and management to mitigate negative impacts on plant biodiversity and ecosystem stability in mountainous landscapes. The results also indicate that in high-elevation ecosystems, even small elevational differences can substantially shape species diversity patterns. When conducting studies involving elevational gradients in similar systems, the selected elevation range should not be excessively wide to capture the ecological nuances of these high-altitude communities.

## Figures and Tables

**Table 1:** Results of model selection. In linear regression models, the response variable was Hill index (*q* = 0, *q* = 1, *q* = 2) of herbs and shrubs. Nine variables including elevation, aspect (ASP), available phosphorus (AP), total phosphorus (TP), total nitrogen (TN), total carbon (TC), ammonium nitrogen (NH_4_^+^-N), nitrate nitrogen (NO_3_^−^-N) and soil pH (pH) were used as explanatory variables. All explanatory variables were standardized to have a mean of zero and standard deviation of one. Only the retained variables in the final best model chosen with lowest AICc are shown.

	Hill Index	Environmental Factors	Estimate	*p*	*R* ^2^
Edge plots					
Shrubs	*q* = 0	Elevation	−0.012	0.042	0.622
		pH	6.224	<0.001	0.622
	*q* = 1	Elevation	−0.014	0.002	0.645
		pH	4.020	<0.001	0.645
	*q* = 2	Elevation	−0.013	0.003	0.571
		pH	3.263	0.003	0.571
Herbs	*q* = 0	Elevation	−0.028	0.034	0.785
		pH	10.693	<0.001	0.785
		TC	1.538	<0.001	0.785
		TP	−0.011	0.004	0.785
	*q* = 1	Elevation	0.013	0.077	0.183
	*q* = 2	Elevation	0.009	0.110	0.152
Interior plots					
Shrubs	*q* = 0	Elevation	−0.034	<0.001	0.592
	*q* = 1	Elevation	−0.030	<0.001	0.673
	*q* = 2	Elevation	−0.027	<0.001	0.646
Herbs	*q* = 0	pH	7.832	0.019	0.298
	*q* = 1	None			
	*q* = 2	None			
Control plots					
Shrubs	*q* = 0	Elevation	−0.109	0.005	0.561
	*q* = 1	Elevation	−0.093	0.008	0.516
	*q* = 2	Elevation	−0.076	0.016	0.458
Herbs	*q* = 0	None			
	*q* = 1	AP	0.229	0.002	0.790
		NH_4_^+^-N	−0.089	<0.001	0.790
	*q* = 2	AP	0.154	0.001	0.958
		NH_4_^+^-N	−0.108	<0.001	0.958
		TC	0.207	0.013	0.958
		TP	0.003	0.022	0.958

Note: “None” indicates that no variables were retained in the final model.

## Data Availability

The datasets generated and/or analyzed during the current study are available from the corresponding author upon reasonable request. The data are not publicly available due to the ongoing nature of the research project and restrictions imposed by the associated research agreements.

## Supplementary Materials

The following supporting information can be downloaded at: https://www.mdpi.com/article/10.3390/plants14223456/s1, Table S1: PERMANOVA results based on Bray–Curtis dissimilarity for differences in shrub and herb species composition among interior (IP), edge (EP), and control (C) plots; Table S2: Results of Non-metric multidimensional scaling (NMDS) testing for an influence of environmental variables on shrub and herb species composition across all plots; Figure S1: Relationships between Hill indices with *q* = 0, 1 and 2 of herb and shrub species and the distance from the plot to firebreak (distance to firebreak).
