# Supplementary material for: Interactive Effects of Firebreak Construction and Elevation on Species Diversity in Subtropical Montane Shrubby Grasslands
Source: Plants (Basel). 2025 Nov 12;14(22):3456. doi: 10.3390/plants14223456 (PMC12656269; doi:10.3390/plants14223456)
Supplement: Supplementary file 1 [file plants-14-03456-s001.zip › plants-3948845-supplementary.pdf]

SUPPORT INFORMATION

Interactive effects of firebreak construction and elevation on species diversity in subtropical montane shrubby grasslands

SUPPLEMENTARY TABLES

Table S1. PERMANOVA results based on Bray–Curtis dissimilarity for differences in shrub and herb species composition among interior (IP), edge (EP), and control (C) plots.

Pairs	Bray-Curtis	F	R^2	p - value
**Shrubs**				
IP vs. EP	0.048	0.513	0.015	0.605
IP vs. C	0.065	26.953	0.490	**0.001**
EP vs. C	0.059	34.183	0.550	**0.001**
**Herbs**				
IP vs. EP	0.058	0.267	0.008	0.844
IP vs. C	0.092	53.520	0.657	**0.001**
EP vs. C	0.089	71.655	0.719	**0.001**

Table S2. Results of Non-metric multidimensional scaling (NMDS) testing for an influence of environmental variables on shrub and herb species composition across all plots.

Environmental factor	R^2	p - value
**Shrubs**		
Elevation	0.976	**0.001**
Aspect	0.046	0.336
pH	0.311	**0.001**
Total nitrogen (TN)	0.528	**0.001**
Total carbon (TC)	0.440	**0.001**
Total phosphorus (TP)	0.995	**0.001**
Available phosphorus (AP)	0.008	0.837
Ammonium nitrogen (NH_4^+-N)	0.397	**0.001**
Nitrate nitrogen (NO_3^--N)	0.522	**0.001**
**Herbs**		
Elevation	0.779	**0.001**
Aspect	0.033	0.463
pH	0.328	**0.001**
Total nitrogen (TN)	0.411	**0.001**
Total carbon (TC)	0.274	**0.001**
Total phosphorus (TP)	0.877	**0.001**
Available phosphorus (AP)	0.003	0.926
Ammonium nitrogen (NH_4^+-N)	0.106	0.062
Nitrate nitrogen (NO_3^--N)	0.254	**0.002**

Note: Nine variables including elevation, aspect (ASP), available phosphorus (AP), total phosphorus (TP), total nitrogen (TN), total carbon (TC), ammonium nitrogen (NH_4^+-N), nitrate nitrogen (NO_3^--N) and soil pH (pH) were fitted to the NMDS ordination to assess their relationships with the ordination axes.

SUPPLEMENTARY FIGURE

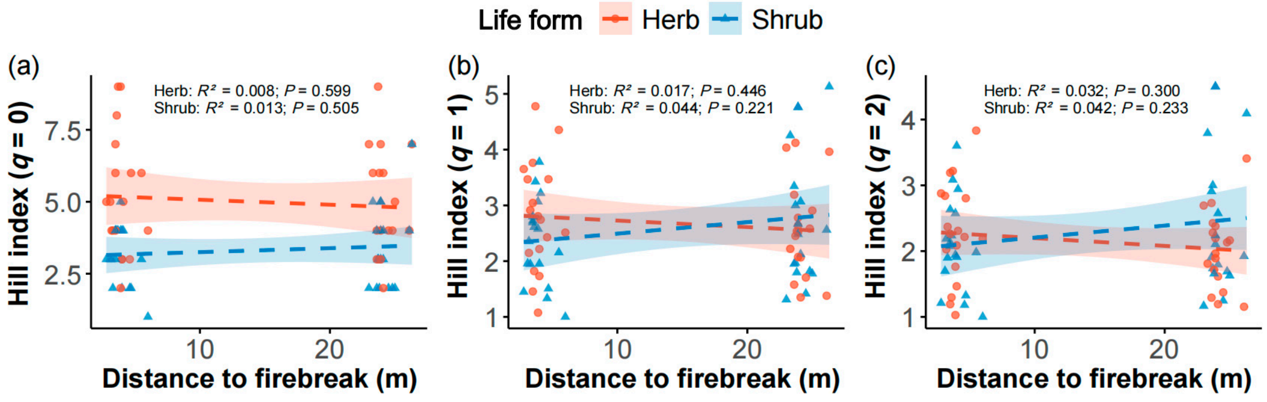

Figure S1. Relationships between Hill indices with q = 0 (a), 1 (b) and 2 (c) of herb (red) and shrub (blue) species and the distance from the plot to firebreak (distance to firebreak). The shaded areas indicate the 95% confidence intervals of the linear regression model. Dashed lines denote non-significant relationships ($P > 0.05$).
